# Evaluation of the Microleakage of Chlorhexidine-Modified Glass Ionomer Cement: An *in vivo *Study

**DOI:** 10.5005/jp-journals-10005-1177

**Published:** 2013-04-26

**Authors:** Sherryl Mary Mathew, Abi Mathew Thomas, George Koshy, Kapil Dua

**Affiliations:** Senior Lecturer, Department of Pediatric and Preventive Dentistry Royal Dental College, Iron Hills, Chalissery, Palakkad-679536 Kerala, India; Principal, Department of Pediatric and Preventive Dentistry, Christian Dental College, Ludhiana, Punjab, India; Professor and Head, Department of Oral and Maxillofacial Pathology Christian Dental College, Ludhiana, Punjab, India; Associate Professor, Department of Conservative Dentistry and Endodontics, Christian Dental College, Ludhiana, Punjab, India

**Keywords:** Dental material, Chlorhexidine, Glass ionomer cements, Microleakage, Sectioning

## Abstract

**Aim:** Recent advances including the incorporation of antibacterial substances, such as chlorhexidine, into restorative materials such as glass ionoer cement (GIC), might alter the physical properties of the material, which might affect the marginal seal of the restorations. Hence, the objective of this study was to compare the marginal sealing ability of GC Fuji IX modified with 1% chlorhexidine diacetate and conventional GC Fuji IX.

**Materials and methods:** Sixty healthy molars were selected from the oral cavities of 30 children. The teeth were divided into two groups: Group I, teeth restored with 1% chlorhexidine diacetate modified GC Fuji IX and group II, teeth restored with GC Fuji IX. The restored teeth were extracted following 4 weeks and immersed in 2% basic fuchsin solution for 24 hours. They were then sectioned and scored under a light microscope of 10 × 10 magnification for dye penetration.

**Results:** On statistical analysis difference between Chlorhexidine-Modified GIC group and GIC group with regard to grade of microleakage was found to be statistically nonsignificant (p = 0.543).

**Conclusion:** Since, addition of 1% chlorhexidine diacetate to GC Fuji IX showed comparable results with regard to microleakage, it can be considered a valuable alternative especially in atraumatic restorative treatment and for general clinical utility in restorative dentistry.

**How to cite this article:** Mathew SM, Thomas AM, Koshy G, Dua K. Evaluation of the Microleakage of Chlorhexidine-Modified Glass Ionomer Cement: An in vivo Study. Int J Clin Pediatr Dent 2013;6(1):7-11.

## INTRODUCTION

The longevity of a restoration is largely dependant on the maintenance of a good marginal seal. Microleakage is defined as ‘the clinically undetectable passage of bacteria, fluids, chemical substances, molecules or ions at the restoration/tooth interface'.^1^

Marginal leakage may be the precursor of secondary caries, may promote tooth discoloration, staining of restorative margins, an adverse pulpal response, postoperative sensitivity and even hasten the breakdown of the filling material.

Most microleakage investigations have been carried out on *in vitro* models with carefully designed simulation of clinical circumstances.

But the conditions vary considerably from those in the oral cavity and consequently there is a need to conduct *in vivo* studies. An outward flow of fluids from freshly cut dentin that may increase wetting of dentin substrate and interfere with the development of adhesive bond to tooth structure.^2^ Other factors include functional stresses, as caused by mastication, the possibility of contamination by saliva or gingival fluids or technical difficulty associated with placement, contouring and finishing of the restoration.

Introduced by Wilson and Kent (1972), glass ionomer cements have been used as excellent dental restorative materials for nearly four decades. It provides slow release of fluoride, which provides a cariostatic action, chemically bonded to enamel and dentin, thereby reducing the need for retentive cavity preparation and is biocompatible with pulpal tissue. However, lack of strength, moisture sensitivity and poor esthetics in conventional (GIC) have limited their use as restorative materials.

This led to the development of a new generation of glass ionomer cements (GC Fuji IX) which were highly viscous, condensable and at the same time provided better esthetics due to the smaller particle sizes. Its additional advantages include its adherence to tooth structure without the need of an additional bond system, adequate strength and fast setting reaction which rationalizes its use in branches of dentistry, such as pediatric dentistry.^3^

Owing to their numerous advantages GIC are fast gaining popularity as the restoration material of choice in minimal intervention approaches, such as atraumatic restorative treatment (ART). Initially, these methods were introduced to target the underprivileged sections of the society but nowadays it is also well accepted by patients with dental anxiety and by children in modern clinical settings, as the sound and pressure caused by rotary instruments is omitted and local anesthesia is not needed. ^4^As the technique is essentially manual, complete elimination of microorganisms are not ensured and a restoration with antibacterial properties would be beneficial. Several hand-mixed conventional GICs have been manufactured specifically for the ART approach.

It is reported in many studies that GIC has antibacterial properties due to the release of fluoride. However, reports have shown that the newer, more viscous GICs release substantially less cumulative fluoride ions than less viscous esthetic restorative GICs and resin-modified GICs. Therapeutic benefit may, therefore, be gained by combining antibacterial agents with glass ionomer material.^5^

The inclusion of antibacterial compounds would (1) eliminate the recurrence of decay around the margins of restorations, (2) inhibit plaque formation on and near the restored surfaces and (3) reduce the number of microorganisms in salivary fluids and oral cavity.

Several substances have been evaluated as possible candidates, but no antimicrobial agent, with the exception of fluoride, has received as much experimental attention as the bis-biguanide chlorhexidine. In the oral environment, persistent reduction of mutans streptococci has been achieved by chlorhexidine varnishes, followed by gels and mouthwashes. When added to dental cements, chlorhexidines have inhibited the growth of bacterial colonies.

Takahashi et al (2006) showed that the concentrations of chlorhexidine release was not dependant on the chlorhexidine content and concluded that 1% chlorhexidine diacetate addition was optimal to give appropriate physical and antibacterial properties to Fuji IX.^6^

Therefore, this current *in vivo* study was designed with the aim to evaluate and compare the marginal sealing ability of modified GIC Fuji IX; 1% chlorhexidine diacetate modified reinforced GIC Fuji IX, when used as restorative materials in primary teeth.

## MATERIALS AND METHODS

### Patient Selection

Ethical approval for the study was granted by the ethical and research committee of the institution. Thirty healthy, cooperative children (10-16 years old) were selected from the Outpatient Department of Pedodontia and Preventive Dentistry, Christian Dental College, Ludhiana. Informed consent was obtained from the parents. Each selected child had at least two sound noncarious primary molars which were liable to exfoliate within the next 6 months, as was determined by the preoperative radiograph.

A conventional powder/liquid type GIC (Fuji IX, GC, Tokyo, Japan) was used as the control material. The experimental GIC was prepared by incorporating chlorhexidine diacetate salt (Smaart Pharmaceuticals, Jalgaon, India) into the powder of the control GIC in a 1/1% w/w ratio.

A split mouth type of study design model was followed.

Group I: Thirty molars with the prepared cavity were restored with 1% chlorhexidine-modified GIC (Fuji IX).

Group II: Thirty molars with prepared cavity were restored with modified GIC (Fuji IX).

### Cavity Preparation and Restoration

Class V cavities, approximately 4 mm wide × 2 mm high × 1.5 mm deep were prepared on the buccal surfaces of non-carious primary molars with no mechanical retention^7,8^ using diamond burs (no. 1 round bur, no. 57 straight fissure bur, and no. 35 inverted cone bur) with contra-angle high-speed air-rotor handpiece with water coolant. All cavosurface angles were kept at 90° without bevel designs. The cavities were prepared keeping a distance of 1 mm from the marginal gingiva following which GC cavity conditioner was applied. The teeth were then restored with GIC (Fuji IX) in bulk placement. The same steps were followed for restoration with 1% chlorhexidine diacetate-modified GIC (Fuji IX). The subjects were recalled after 1 month.^8^ Both the restored teeth were extracted on the same day, taking care to avoid any stresses that compromised the seal of the restoration with the cavity preparation. The extracted teeth were cleaned, all the tooth surfaces except the restoration and a 1 mm zone adjacent to its margins were covered with two coats of nail varnish. The root apices, if any, were sealed with sticky wax .The coated teeth were immersed in a 2% aqueous solution of basic fuschin dye for 24 hours at room temperature. After peeling of the coatings, the teeth were thoroughly washed, dried, embedded in self-curing acrylic resin and sectioned into two halves buccolingually in an occlusoapical direction through the middle of the restoration.

### Microscopic Examination and Scoring

Each section thus prepared was inspected using a ligh microscope (Motic-MC 2000 B1 series) with video outpu device (monitor with Windows XP supported hardware) to assess the dye penetration at the margins of the restoration The microleakage was observed at a magnification o 10 × 10. A Motic MC camera software was used to capture the images.

### Scoring Criteria

*Score 0:* No dye penetration.

*Score 1:* Dye penetration between the restoration and the tooth into enamel only.

*Score 2:* Dye penetration between the restoration and the tooth in enamel and dentin.

*Score 3:* Dye penetration between the restoration and the tooth in the pulp chamber.

The scores were tabulated, interpreted, and the resultant findings were statistically analyzed using the Chi-square test.^7^

## RESULT

### Group I

**Fig. 1:**
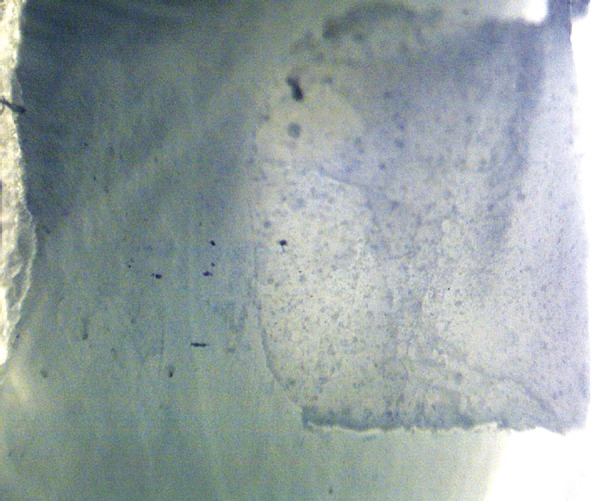
Score 0: No dye penetration

**Fig. 2:**
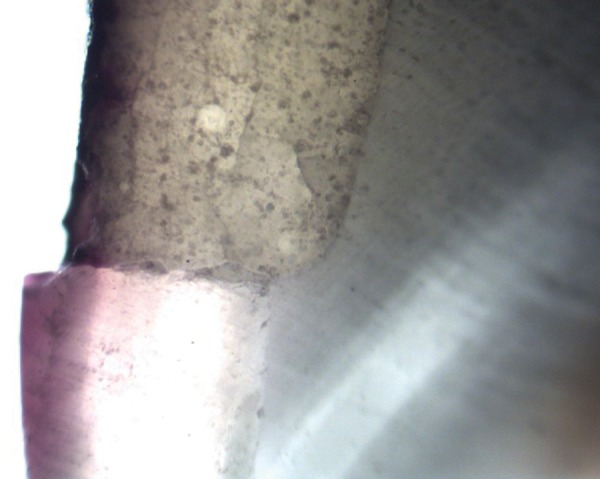
Score 1: Dye penetration between the restoration and the tooth into enamel only

**Fig. 3:**
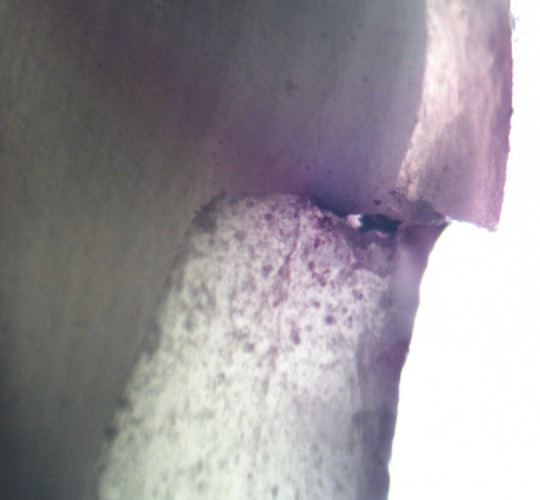
Score 2: Dye penetration between the restoration and the tooth in enamel and dentin

Out of 30 samples studied for microleakage using 1% chlorhexidine diacetate-modified GIC (Fuji IX) 26 samples showed no dye penetration (86.7%) (Fig. 1: Score 0–no dye penetration), one sample showed dye penetration in enamel (3.3%) (Fig. 2: Score 1–Dye penetration between the restoration and the tooth into enamel only), and three samples showed dye penetration in enamel and dentin (10.00%) (Fig. 3: Score 2–Dye penetration between the restoration and the tooth in enamel and dentin) as observed in Table 1. However, in this group none of the samples showed dye penetration in pulp.

### Group II

Out of 30 samples studied for microleakage using Fuji IX, 25 samples showed no dye penetration (83.3%), three samples showed dye penetration in enamel (10.00%) and two samples showed dye penetration in enamel and dentin (6.7%), as observed in Table 1. However, in this group none of the samples showed dye penetration in pulp.

**Table 1:** **Table 1:** Comparison of microleakage scores of 1% chlorhexidine diacetate-modified GIC (Fuji IX) with that of modified GIC (Fuji IX)

*Grade of* *Microleakage*	*Group I* *CMGIC*	*Group I* *GIC*	Total
0	26	25	51
1	1	3	4
2	3	2	5
Total	30	30	60

*X^2^*= 1.220; df = 2; p = 0.543; Not significant

CMGIC: Chlorhexidine-modified glass ionomer cement

The Chi-square test was used to predict the comparison in dye penetration between the two groups. After statistical analysis, difference between CMGIC group and GIC group with regard to grade of microleakage was found to be statistically nonsignificant (p = 0.543).

## DISCUSSION

These results are in harmony with the fact that Fuji IX has a coefficient of thermal expansion comparable to that of the tooth. The scores obtained in this study validated a previous study done by Castro and Feigel who concluded that this new generation of GICs had significantly less leakage than similar cavities filled with conventional GIC.^3^

Frankenberger et al (1997) and Berg (1988) reported that Fuji IX sets faster and are of higher viscosity because of finer glass particles, anhydrous polyacrylic acids of higher molecular weight and a high powder to liquid ratio.^9^ These properties may be responsible for GC Fuji IX restorations showing good marginal seal.

The results revealed that group I scored slightly higher in terms of dye penetration scores but there was no statistically significant difference in the mean microleakage values between group II-modified GIC (Fuji IX) and group I (1% chlorhexidine diacetate-modified Fuji IX). This confirms our hypothesis that the microleakage values obtained in the 1% chlorhexidine diacetate-modified GIC group are comparable with the Fuji IX group. This could have ensued because the minor amount of chlorhexidine did not hinder the formation of bond of the glass ionomer with the tooth structure. Since, there have been very few studies evaluating the microleakage of chlorhexidine-modified GIC, the results could not be compared.

Condensable GICs, more properly termed high viscosity GICs were developed early in the 1990's largely as a response to the need for filling materials in the atraumatic restorative therapy technique. These high viscosity GICs have greater flexural strength and better wear resistance, largely due to the smaller mean particle size and increased viscosity.^9^ Thus, Fuji IX, a new high density GIC was believed to offer significant benefits when used in conjunction with minimal intervention techniques like atraumatic restorative treatment in the management of early childhood caries and patients who are unable to tolerate conventional treatment.^10^

However, reports have shown that the newer, more viscous GICs release substantially less cumulative fluoride ions than less viscous esthetic restorative GICs and resin-modified GICs.^5^

A study by Marsh and Bradshaw, 1990 showed that 1 mmol/l fluoride [18.87 ppm (microgram/ml) F] combined with a moderated low pH can prevent *Streptococcus mutans*.^11^ Fuji IX has been reported to release approximately 10 ppm fluoride during 48 hours.^12^ However, such amounts of fluoride are too small to exhibit antibacterial effects as demonstrated in their agar diffusion tests.^6^

The combination of antibacterial agents with restorative materials and specifically chlorhexidine has been investigated previously.^6,13^

Chlorhexidine is a bis-biguanide and is strongly basic, containing two positive charges. It is, therefore, described as dicationic and has an affinity for negatively charged surfaces, such as bacterial cell walls, extracellular polysaccharides of bacterial origin, hydroxyapatite, pellicle, plaque, salivary mucin and oral mucosa.

Chlorhexidine inhibits the metabolism of *S. mutans* by suppressing enzymes like glycosyltransferase as well as the phosphoenolpyruvate – phosphotransferase system. The bacteriostatic spectrum of chlorhexidine has been determined: There was a wide spectrum of activity with Gram-positive cocci being especially sensitive (MIC, 0.19 to 2.0 μg/ml). Exposure of suspensions of various bacterial species to chlorhexidine (0.02%) for 10 minutes at room temperature reduced the viable organisms by about 99.99% in most instances.^14^ However, it is the persistent effect or substantivity of chlorhexidine that appears to be responsible for its success as an antimicrobial and antiplaque agent. In this aspect it appears unrivalled at present.

The salts, most commonly used, are chlorhexidine digluconate, chlorhexidine dihydrochloride and diacetate. Of the following, the chlorhexidine digluconate cannot be isolated in substance and is stable only in diluted solutions.^5^ Besides, chlorhexidine gluconate when added to glass-ionomer does inhibit the growth of *S. mutans* but it also results in a decrease in the physical properties of the material which may be related to the fact that it is a liquid and leaches out more rapidly than the powder or diacetate form of chlorhexidine.^15^

Both chlorhexidine salts, i.e. chlorhexidine dihyrochloride and chlorhexidine diacetate are powdery compounds, which can be easily mixed with GIC powder.^6^ It has been stated that the chlorhexidine diacetate powder has a low solubility in water, 1.9%w/v, and thus the enclosed particles are released during the deterioration of the material.^14^ To add to its benefits, chlorhexidine diacetate is a more stable material, not prone to decomposition, and can be easily blended with GIC.^5^

The antimicrobial activity of chlorhexidine added to GIC can be increased significantly when added above concentrations of 5% but the material tends to deteriorate rapidly for it to be used as a restorative material.^5^ In addition chlorhexidine does not contribute to the formation of the glass ionomer network, and therefore, high amounts of chlorhexidine would weaken the scaffold and compromise the mechanical properties of GICs. Besides, chlorhexidine additives are classified as harmful and rather toxic [LD50 (mouse, oral)-2,515 mg/kg] and hence it is preferable to keep the amount of chlorhexidine as low as possible.^5^ This is evident in the studies carried out by Takahashi et al (2006) and Frencken et al (2007) which led to the conclusion that addition of 1% chlorhexidine diacetate is optimal to give appropriate antibacterial, physical and bonding properties to Fuji IX.

Thus, taking into consideration the improved antibacterial characteristics of chlorhexidine-modified GIC (Fuji IX), it can be concluded that combined with the favorable microleakage results as obtained in the present *in vivo* study there is scope for further research on the clinical characteristics of this material. Since, the antibacterial properties have been shown to have promising results, further studies are warranted to establish the durability of this material. The results of this *in vivo* study can be considered promising for the use of this material in conjunction with the principles of minimal intervention techniques, such as atraumatic restorative treatment and thus can enable the provision of treatment to the lesser privileged sections of society.

## CONCLUSION

1. Incorporation of 1% chlorhexidine diacetate salt to GIC (Fuji IX) does not seem to alter substantially the physical properties of the restorative material such as microleakage.
2. Due to their enhanced antibacterial properties and comparable marginal sealing abilities 1% chlorhexidine diacetate-modified GIC can be considered a valuable alternative, especially in conjunction with minimal invasive techniques, as well as for general clinical utility in pediatric dentistry.
